# Enhancing Hepatitis B case finding through tech-enhanced community pharmacy: Results from a trial program in Vietnam

**DOI:** 10.1371/journal.pgph.0007137

**Published:** 2026-08-26

**Authors:** Khue Nguyen Y Ha, Sinit Mehtsun, Boon-Leong Neo, Tuan Minh Nguyen, Tien Ho, Hang Viet Dao, Josselyn Neukom

**Affiliations:** 1 Public Health Division, SwipeRx, Ho Chi Minh City, Vietnam; 2 Gilead Sciences, San Francisco, California, United States of America; 3 Gilead Sciences, SingaporeSingapore; 4 Internal Medicine Faculty, Hanoi Medical University, Hanoi, Vietnam; Universidad Autonoma de Baja California, MEXICO

## Abstract

Hepatitis B virus (HBV) remains a major public health challenge in Vietnam. Barriers include limited awareness and access to screening as well as out-of-pocket diagnosis and treatment costs. Point-of-care rapid diagnostic tests (RDTs) offer an opportunity to expand screening and linkages to diagnosis and care. This intervention assessed technology-enhanced community pharmacies ability to provide HBV screening and find cases in Vietnam using digital monitoring and cross-sectional digital survey data. Between February 2024 and March 2025, SwipeRx partnered with the Vietnamese Pharmaceutical Association and the commercial supplier of Determine HBsAg-RDT to implement a trial program including digital campaigns, accredited digital training, in-pharmacy and mobile coaching, and supplier linkages. Monitoring and cross-sectional digital survey data were used to assess changes in HBV knowledge and practices at the pharmacy. Among 917 pharmacists who completed the accredited online training, 117 pharmacies with at least 1 trained pharmacist received coaching and procured 5,900 HBsAg-RDTs, with 48% placing repeat orders. During the same six-month period, 84% (98/117) of pharmacies identified 270 pharmacy clients who tested positive out of 2,840 unique clients screened using HBsAg-RDT prior to referral to a nearby health facility for confirmatory HBV diagnosis. Pharmacists who completed the digital training and were engaged with digital campaigns and coaching were 2.83 times more likely to stock HBsAg-RDT and reported significantly higher HBV-related knowledge and confidence compared to non-engaged pharmacists. This research demonstrates how technology-enabled training and pharmacy engagement can facilitate community access to new health products and services, including HBV screening. Optimizing test packaging, pricing, and pharmacy client demand may further expand access to and uptake of HBV screening through community pharmacies in future.

## Introduction

Chronic infection with Hepatitis B virus (HBV) remains a major global public health challenge, affecting an estimated 254 million people worldwide, with substantial gaps in diagnosis and treatment [1]. In Vietnam, an estimated 9.6 million people are living with HBV [2–4]. Of these, only 10% have been diagnosed, and only 3% are receiving treatment [1,5,6]. The annual cost of outpatient consultation, diagnosis, and treatment for HBV is approximately 16 million Vietnamese dong (US dollar $695), often paid partially or fully out-of-pocket because insurance coverage only applies in medically indicated cases identified by doctors at health facilities covered by the insurance system [7]. Considering the average monthly income per capita among adults in Vietnam is 4.25 million Vietnamese dong (US dollar $184), without full insurance coverage, the costs of HBV diagnosis and care remain substantial barriers [8]. Access barriers are further compounded by the reliance on larger public health facilities as a source of HBV testing, which creates challenges related to transportation and logistics [5,8]. Before the intervention described in this paper, the only commercial supplier of rapid diagnostic tests for detection of hepatitis B surface antigen (HBsAg-RDT) approved for use without laboratory facilities reported no sales to community pharmacies in Vietnam. All Determine HBsAg-RDT were sold to public and private hospitals. In summary, limited access to community-based HBV screening, combined with logistical challenges to public-hospital HBV service uptake for some people in need of HBV care, represented a major barrier to finding and treating missing cases of HBV in Vietnam at the time this trial program was designed.

Globally, community pharmacy-based screening initiatives for HBV and other infectious diseases have proven effective, with published studies reporting substantial case detection results, including HBsAg positive rate of up to 17.5% in pharmacy-based screening settings [9–11]. In line with World Health Organization recommendations, point-of-care RDTs are a practical tool for scaling HBV screening [12–14]. Despite this recommendation, prior to this trial program, community pharmacies had not been included in Vietnam’s national HBV programming [7]. Despite the Vietnamese Ministry of Health’s guidelines endorsement of HBV screening at community level, as of 2023, when the trial program described in this manuscript was designed, pharmacies were not involved in the national program. Determine (manufactured by Abbott Diagnostics, imported and distributed by Delta supplier) was the only HBsAg-RDT registered with local authorities for use outside of laboratory-equipped health facilities in Vietnam. When the trial program began, Abbott’s supplier Delta sold Determine HBsAg-RDT in 100-test packs to public and private hospitals only.

Between February 2024 and March 2025, SwipeRx partnered with the Vietnamese Pharmaceutical Association and Delta to implement a technology-enhanced trial program aimed at strengthening pharmacy capacity to offer HBV screening and referral services. Using digital campaigns and an accredited digital training offered through the SwipeRx pharmacy-focused app as well as in-pharmacy coaching, and direct supplier linkages, the program motivated retail pharmacies to offer HBV screening and referral services in the three cities and one province: Hanoi, Da Nang, Ho Chi Minh City and Hung Yen.

The aim of this research was to assess a technology-enhanced model for HBV screening, referral, and case finding through community pharmacies in urban and rural areas of Vietnam and to evaluate differences in pharmacists’ knowledge, attitudes, and practices between “engaged” pharmacists trained and coached through SwipeRx and non-engaged pharmacists using digital monitoring and digital survey data.

## Materials and methods

### Ethics statement

This study protocol received ethical approval from the Ethics Committee of the Hanoi University of Public Health, Vietnam (approval number 37/2025/YTCC-HD3; approved on 27/02/2025). Participation in the digital survey was voluntary. At the start of the survey, all participants were presented with an electronic informed consent form. Only participants who provided written electronic consent (via checkbox confirmation) were able to proceed and complete the questionnaire. No personal identifying information was collected, and all data were fully anonymized prior to analysis.

### Study design

A prospective study design combining digital monitoring and cross-sectional digital survey data was employed to assess a technology-enhanced intervention to improve pharmacists’ knowledge, attitudes, and practices related to HBV screening in urban and rural Vietnam.

### Intervention

A combination of pharmacist-focused digital campaigns and an accredited online training, together with in-pharmacy post-training support, was used to introduce HBsAg-RDTs into community pharmacy practice in Vietnam. In February 2024, the Determine HBsAg-RDT was the only RDT approved by Vietnamese regulatory authorities for use outside health facilities with laboratory capacity [15,16]. SwipeRx, with support from Gilead Sciences, implemented a multi-component intervention including:

- Digital awareness campaigns deployed through the SwipeRx app newsfeed and social media channels during February–March 2024 and June 2024. The campaigns included interactive games, polls, and posters designed to raise awareness about HBV burden in Vietnam and to encourage pharmacies to stock Determine HBsAg-RDT by procuring through Delta—the commercial supplier offering pricing and delivery terms specifically negotiated for trained pharmacists.
- Accredited digital training in April 2024, organized in partnership with the Vietnam Pharmaceutical Association. The 8-hour accredited digital training offered through the SwipeRx platform was designed to equip pharmacy professionals with the knowledge and skills needed to offer HBV screening and referral services using the Determine (finger prick, blood-based) HBsAg-RDT.
- Post-training coaching: using both mobile (through the Zalo messaging platform) and face-to-face methods. Trained pharmacists who procured Determine HBsAg-RDT received countertop standees to prompt uptake of HBV testing among unvaccinated adult pharmacy clients.
- In May 2024, the trial program team advocated with the manufacturer to import the smaller, 20-test package of Determine HBsAg RDT, following resistance from pharmacies to stocking the larger 100-test package initially due to relatively high upfront investment requirements for a product newly introduced into community pharmacies, relatively low client demand for HBV testing, and concerns about product expiration before use of a relatively large pack size.

Pharmacies were voluntarily recruited into the trial program through digital outreach conducted through the SwipeRx platform and pharmacy visits conducted by the program team. Pharmacies with one or more pharmacists who completed the digital training, received post-training coaching, and subsequently procured and stocked Determine HBsAg-RDT, were included in this analysis as “SwipeRx-engaged.”

### Cross-sectional digital survey

Thirteen months after the launch of the intervention, a cross-sectional quantitative digital survey was conducted via the SwipeRx mobile app. Data collection was completed in 14 days between 10 to 26 March 2025. Eligible survey respondents were pharmacy professionals aged 18 or older, registered on SwipeRx with complete profile information, involved in dispensing and counseling practices in retail pharmacies in Hanoi, Ho Chi Minh City, Da Nang, or Hung Yen province. Only one survey respondent per pharmacy was allowed.

#### Sample size calculation.

Assuming a correct-response rate of 70% among engaged pharmacists (p_1_ = 0.7) and 50% among non-engaged (p_2_ = 0.5), with a two-sided α of 0.05 (*Z*_1-_⍺_*/2*_ = 1.96), a power of 80% (*Z*_1-*β*_ = 0.84), and a ratio of 1:2 between groups (r = 0.5), the required sample size per group was calculated as follows [17,18]:


$$\begin{array}{c} n_{\text{2}}=\;\frac{{\left(Z_{\text{1}-\alpha /\text{2}\;}+Z_{\text{1}-\beta }\right)}^{\text{2}}\times \;[\frac{p\text{1}(\text{1}-p\text{1})}{r}+p\text{2}(\text{1}-p\text{2})]}{{\left(p\text{2}-p\text{1}\right)}^{\text{2}}}\;,\;n_{\text{1}}=r\;\times \;n_{\text{2}} \end{array}$$

Based on this calculation, the minimum required sample size was 66 respondents from engaged pharmacies and 132 from non-engaged pharmacies.

#### Survey development and distribution.

The digital survey instrument included 48 questions and was developed in English prior to translation to Vietnamese. Domains included demographics, client profile, HBV-related knowledge and training, attitudes towards HBsAg-RDTs, screening, referral and counseling practices, and perceived barriers. Prior to launch, the survey was tested with 3 pharmacists through the SwipeRx app to check accessibility, clarity, and estimated completion time. Digital survey recruitment used app push notifications, pop-up invitations, SMS links, phone calls, social media and mobile channels as well as pharmacy visits. Participation was voluntary, and informed consent was required before pharmacy professionals completed the survey in the SwipeRx app.

### Data analysis

Digital monitoring data collected through the SwipeRx app assessed exposure and engagement with digital campaigns as well as post-training changes in knowledge, and test procurement and use practices. To assess changes in pharmacist knowledge, pre- and post-training assessment scores were compared using the paired t-test. The impact of post-training mobile and in-pharmacy coaching was assessed by the number of Determine HBsAg-RDTs procured and used by participating pharmacies. In addition, the number of HBV-positive cases identified through pharmacy-based screening was tracked monthly through in-pharmacy visits conducted by the SwipeRx Field Officers.

Digital survey responses collected via Qualtrics were cleaned to remove duplicates or responses with less than 80% survey questions answered. STATA 17 software (StataCorp, College Station, TX, USA) was used for analysis. Descriptive statistics were produced for all variables. Knowledge, attitudes, and practices were compared between engaged and non-engaged pharmacies, as well as between urban and rural respondents. Chi-square tests were used to compare categorical variables when expected cell counts were ≥ 5; otherwise, Fisher’s exact tests were applied. Continuous variables were compared using Mann-Whitney U test when data were not normally distributed. Multiple linear regression analysis with robust standard errors was performed to identify factors associated with HBV-related knowledge among sampled pharmacists, including urban/rural location, engagement status (engaged vs. non-engaged), age, and pharmacy professional type (pharmacist vs. non-pharmacist including pharmacy owner, pharmacy manager and pharmacy assistant). A p-value of <0.05 was considered statistically significant for all analyses.

### Intervention and data collection timeline

**Fig 1:**
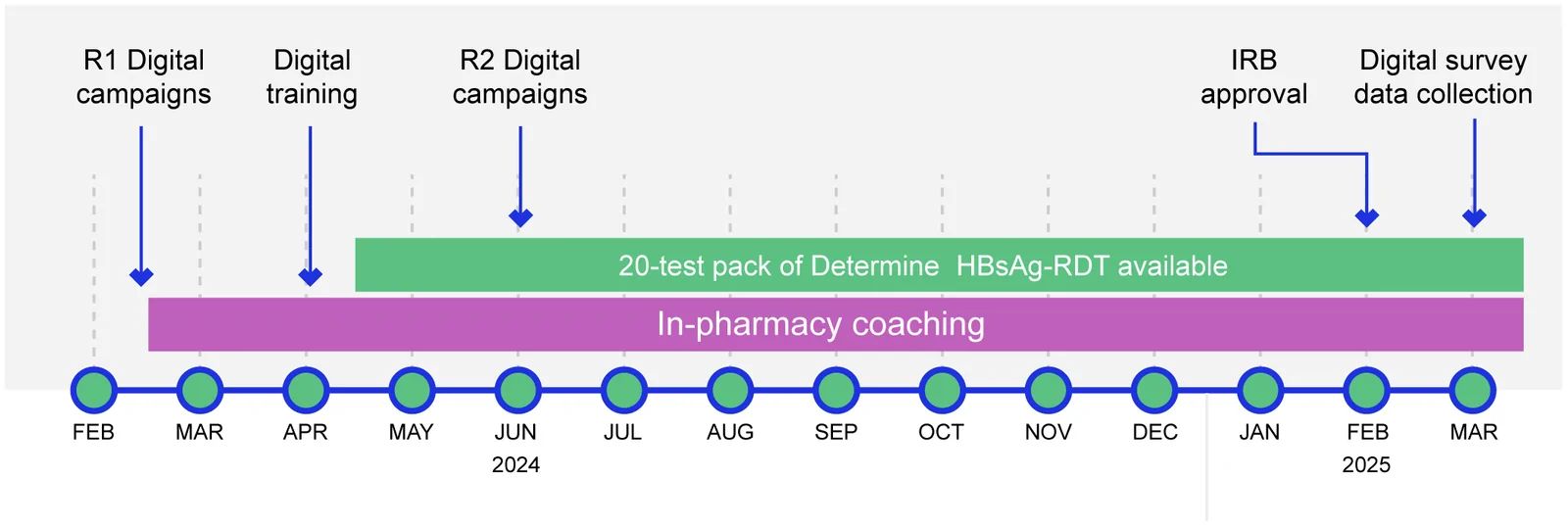
Intervention and digital survey timeline. Timeline illustrating the deployment of digital campaigns round 1 (R1) and round 2 (R2), accredited digital training, availability of Determine, a rapid diagnostic test for detection of hepatitis B surface antigen (HBsAg-RDT) packs for pharmacy stocking, Institutional Review Board (IRB) approval, and digital survey deployment between February 2024 to March 2025.

Fig 1 presents the timeline, which illustrates the sequencing of the digital survey and other trial program activities.

## Results

### Intervention outcomes

Between February 2024 and March 2025, the trial program achieved several key activities related to pharmacy access to HBV screening and referrals in three cities and one rural province. The digital campaign achieved notable increases in reach and engagement when the second round is compared to the first round. The first round (deployed over 3 weeks in February and March 2024) reached 1,251 unique SwipeRx user accounts and generated 875 engagement points defined as likes, shares, or comments on campaign content. The second round was deployed over four weeks in June 2024 and reached 3,603 unique SwipeRx user accounts and generated 3,108 engagement points, which was more than 3.5 times higher than in the first round.

An 8-hour accredited digital training for pharmacists was organized in partnership with the Vietnam Pharmaceutical Association in April 2024. Out of 1,249 pharmacy professionals who attended the training, 1,000 completed the post-training assessment questions, and 917 participants successfully answered 70% or more of the questions correctly required for continuing medical education (CME) certificate eligibility in Vietnam. Of these, 476 of the trained pharmacists who received CME certificates were from the four focus cities and provinces for the trial program. Post-training HBV knowledge increased by 28% among trained pharmacists compared to pre-training levels: from 68% to 96%. There were no significant differences in post-training HBV knowledge between urban and rural pharmacists (95.6% vs. 95.5%, p > 0.05). Reported ability to correctly use Determine HBsAg-RDT increased by 39% among trained pharmacists.

Through post-training coaching, 117 pharmacies were motivated to procure Determine HBsAg-RDT from the commercial supplier and offer HBV screening to unvaccinated adult clients. Coaching outreach through digital and in-pharmacy channels focused on i) re-emphasizing key points from the training including Vietnam’s HBV burden and the need for community screening to increase case finding; and ii) helping pharmacists connect with the commercial supplier to procure at negotiated prices and delivery terms. Between October 2024 and March 2025, 49 pharmacies in Hanoi, 25 in Ho Chi Minh City, 25 in Da Nang, and 16 in Hung Yen, as well as two in non-focus provinces, procured 5,900 HBV screening tests from the commercial supplier. Among these, 48% procured Determine HBsAg-RDT tests two or more times during the same six months. During the 6-month trial program, 2,840 of 5,900 procured Determine HBsAg-RDTs were used to screen 2,840 unique pharmacy clients. 84% (98 out of 117) of the engaged pharmacies referred 270 clients who were subsequently confirmed with HBV diagnosis at public or private health facilities, representing 9.5% positivity. To verify HBV-positive case findings, pharmacists sent photos of positive HBsAg tests to the team members responsible for pharmacy coaching activity under the trial program. After clients were referred for confirmatory testing, pharmacists used mobile follow-up to receive the client’s confirmed testing result from the health facility. At the end of each month, trained pharmacists sent a zalo message with all confirmed HBV cases against positive HBsAg test results. Table 1 presents the Determine HBsAg-RDT procurement, use, and confirmed HBV-positive case finding data by location between October 2024 and March 2025. The number of tests procured and used varied by location given the different number of pharmacies with a trained pharmacist as well as differences in procurement timing within the 6-month monitoring period. The proportion of procured tests used at pharmacies in the three cities and focus province Hung Yen was between 42–56%. While the positivity rate was slightly higher in Hanoi, Da Nang and Hung Yen (11–13%) compared to Ho Chi Minh City (7%), without more detailed analysis of client demographics it is difficult to interpret these findings.

**Table 1:** Determine HBsAg-RDTs procurement, administration, and HBV case finding by pharmacy location.

Pharmacy Location	HBsAg-RDTs Procured, n	HBsAg-RDTs Used, n	HBV-Positive Cases Confirmed, n
**Hanoi**	1,800	760	95
**Ho Chi Minh**	2,340	1,100	80
**Da Nang**	560	280	33
**Hung Yen**	1,000	560	60
**Other**	200	140	2
**Total**	**5,900**	**2,840**	**270**

### Digital survey findings

A total of 210 pharmacy professionals completed a digital survey deployed through the SwipeRx app in Vietnam, with a mean age of 36 years. Slightly more than one-third (37%) of survey respondents were affiliated with pharmacies with a pharmacist trained and coached through SwipeRx, exceeding the targeted sample size for this segment by 33%. The majority of respondents (79%) were based in urban areas, consistent with the geographic focus of the intervention in three urban areas and one rural province. In terms of professional background, 71% of survey participants identified pharmacist as their primary professional role. The detailed demographic characteristics of survey respondents are presented in Table 2.

**Table 2:** Demographic characteristics of digital survey respondents.

	Total (N = 210)	Engaged (n = 77)	Non-engaged (n = 133)	p-value
**Age (years), mean (SD)**	35.98 (8.16)	36.64 (7.71)	35.60 (8.41)	0.3771
**Urban areas, n (%)**	166 (79%)	59 (76.6%)	107 (80.5%)	0.511
**Pharmacy professional type, n (%)**				
Pharmacist	150 (71.4%)	46 (59.7%)	104 (78.2%)	0.004
Others^*^	60 (28.6%)	31 (40.3%)	29 (21.8%)	

* including pharmacy assistant, pharmacy manager, and pharmacy owner

### HBsAg screening & referral access at pharmacies

Overall, 59% of survey respondents reported stocking HBsAg-RDTs. Respondents from engaged pharmacies were significantly more likely to report stocking HBsAg-RDTs compared to respondents from non-engaged pharmacies (prevalence ratio 2.83, 95% CI 2.25–3.56; p < 0.001). Among pharmacies stocking HBsAg-RDTs, 57% reported screening 1–5 clients per month. Counseling practices varied, with most surveyed pharmacists reporting that they spent 5–15 minutes on HBV counseling. Engaged pharmacists reported significantly greater confidence in counseling on HBV risks and benefits and in the test accuracy (98% vs. 59% and 88% vs. 48%, both p < 0.001). 96% reported referring clients with positive HBV results to public health facilities, and 45% to private health facilities, reflecting a mix of referral practices.

Survey respondents offered practical suggestions to improve the uptake of HBV screening services at pharmacies in future, including smaller packaging options—such as boxes containing 5 or 10 tests instead of the 20 and 100-test pack sizes available for pharmacy procurement at the time the trial program was implemented—to reduce upfront investment requirements by pharmacists and minimize expiration risks. In addition, HBsAg- RDT packages including all required materials (e.g., lancets, buffer solution, alcohol swabs) with the tests have potential to facilitate easier use at community pharmacies. Lastly, surveyed respondents recommended reducing the retail price of the test.

### HBV training experience & knowledge among pharmacists

All respondents from engaged pharmacies had completed HBV training compared to 29% of non-engaged (p < 0.001). Respondents from engaged pharmacies also demonstrated significantly higher HBV-related knowledge, with mean scores 4.35 points higher than those of non-engaged counterparts (8.45 vs. 4.10 out of the maximum 12 points, p < 0.001). Specifically, knowledge score related to HBsAg-RDTs was 2.58 among engaged compared to 1.38 among non-engaged (out of a maximum 4 points, p < 0.001). Correct responses to knowledge-based survey questions were consistently higher among engaged pharmacies across all the domains assessed: HBV burden (87% vs. 22%), correct use of the Determine HBsAg-RDT (96% vs. 50%), referral practices (68% vs. 21%), and awareness of the link between HBV and liver cancer risk (92% vs. 20%) (all p < 0.001). Notably, there was no significant difference in HBV-related knowledge levels between engaged pharmacists in urban and rural areas (8.5 vs. 8.3 out of the maximum 12 points, p > 0.05).

Multiple linear regression analysis found that engagement level was independently associated with higher total HBV-related knowledge scores after adjusting for urban/rural location, age, and pharmacy professional type (β = 4.33, 95% CI 3.80–4.86, p < 0.001). The survey respondent’s location, age, and pharmacy professional type were not significantly associated with HBV knowledge levels. Results of the multiple linear regression analysis are presented in Table 3.

**Table 3:** Multiple linear regression analysis: Factors associated with HBV-Knowledge.

Variable	β coefficient	95% confidence interval	p-value
Engaged vs. non-engaged	4.33	3.80–4.86	<0.001
Urban vs. rural	0.23	−0.52–0.98	0.541
Age (years)	0.01	−0.02–0.04	0.520
Pharmacist vs. non-pharmacist^*^	−0.20	−0.76–0.36	0.491

*Non-pharmacists include pharmacy assistant, pharmacy owner, and pharmacy manager

### Attitudes related to HBV screening among pharmacists

Attitudes regarding HBV screening varied notably between respondents from engaged and non-engaged pharmacies. Pharmacists from engaged pharmacies reported recognizing multiple advantages of the Determine HBsAg-RDT, citing its quick testing process, accuracy, and ease of use, while non-engaged pharmacists reported low customer demand, limited awareness of the test’s availability, and uncertainty about regulatory requirements as key barriers to stocking the test. When asked about broader challenges to offering HBV screening and counseling, insufficient client demand was the most frequently cited barrier by pharmacists from both segments (79% of engaged vs. 59% of non-engaged, p < 0.05). Additional barriers cited included the cost of the test and time constraints associated with test administration and counseling (31% vs. 29%, p > 0.05). Limited staff knowledge and perceived policy restrictions were more commonly cited by non-engaged pharmacies (44% vs. 13%, p < 0.001).

Across all pharmacists surveyed, more than half believed that pharmacies should offer HBV rapid tests, and approximately two-thirds agreed that HBV screening should be widely accessible through community pharmacies. There was no statistically significant difference in pharmacist attitudes related to HBV screening when comparing urban and rural locations.

## Discussion

Both digital monitoring and survey data identified higher HBV screening and referral knowledge and practices among pharmacists engaged with digital training and mobile as well as in-pharmacy coaching. These results demonstrate the ability of training and coaching through a pharmacy-focused digital platform to facilitate introduction of a new product and service at community pharmacies. After training 917 pharmacy professionals through an accredited webinar in a single day, the trial program was able to motivate trained pharmacy professionals to stock Determine HBsAg-RDT and offer HBV screening and referral services to clients during a six-month trial program period. Monitoring data collected immediately following the online training and survey data collected 13 months after the trial program began revealed improvements in HBV knowledge among urban as well as rural pharmacists engaged with training and post-training coaching activities. A combination of mobile and in-pharmacy post-training coaching successfully motivated pharmacy professionals at 117 community pharmacies in three cities and Hung Yen province to stock and use Determine HBsAg-RDT with unvaccinated adult clients visiting their pharmacies. During six months, over 80% of pharmacies offering HBV screening services found one or more positive cases demonstrating a correlation between improvements in pharmacy capacity, screening test access and HBV case finding. While the positivity rate was slightly higher in northern cities and Hung Yen province (11–13%) compared to Ho Chi Minh City (7%), pharmacies in urban as well as rural areas used a similar proportion of procured tests to offer HBV screening to their customers. This indicates willingness both among pharmacy staff and their customers in multiple locations to offer and accept HBV screening using pharmacist-assisted use of a finger-prick, blood based RDT.

These results highlight the potential role of community pharmacies to support the World Health Organization’s recommendation for “task sharing of point-of-care testing with non-lab personnel” by training, monitoring and supervising pharmacists to stock and use finger-prick, blood-based rapid screening tests to increase case finding [19]. These results are consistent with experience in Vietnam and other countries, where pharmacies and other community-level healthcare workers have been successfully leveraged to expand access to screening for HIV, hypertension and diabetes screening using a variety of test types as a gateway to increasing diagnosis and treatment rates [9–11,20–29]. In Kenya pharmacy-based HIV rapid testing programs demonstrated that community pharmacies can expand access for high-risk populations who may not otherwise utilize traditional healthcare facilities [27,28]. Similarly, research in other settings has shown the importance of community pharmacies offering hypertension and diabetes screening together with referral services as a path to increasing case detection [24,29]. Evidence described in this manuscript is consistent with previously published literature and extends this evidence by demonstrating the ability of trained pharmacists to contribute to HBV case finding using finger-prick, blood based RDT.

The lack of statistically significant differences in pharmacist knowledge, attitudes, or practices among rural compared to urban pharmacists engaged by the intervention in Vietnam, suggests that technology-enabled pharmacy engagement model may have potential to contribute to national HBV program goals across diverse geographic settings. These findings emphasize the potential value of expanding community access to HBV screening through pharmacies together with public and private health facilities.

Ongoing barriers to HBV screening through community pharmacies include limited client demand of HBV risk as well as limited awareness of approved HBsAg-RDTs capable of being used outside of laboratory-equipped health facilities. In addition, appropriately packaged for individual use and lower priced HBsAg-RDT packaging can facilitate access to and uptake of HBV screening through community pharmacies in future. While the intervention’s successful negotiation for the importation of a 20-test pack of Determine HBsAg-RDT was key to convincing more community pharmacies to stock Determine HBsAg-RDT and offer HBV screening services, a smaller pack size containing all testing equipment needed for a single customer and a lower price point per test would facilitate greater uptake at the community level in Vietnam and similar contexts. Given Vietnam’s commitment to Universal Health Coverage and ongoing efforts to optimize social health insurance coverage, future programs should also advocate for pharmacy use of HBV RDT to be reimbursed by Vietnam’s national insurance [30].

Overcoming these barriers in the future requires support from HBV screening test manufacturers and importers to ensure convenient and affordable access to screening tests designed for use at the community level. In addition, greater public awareness regarding the risks of HBV among unvaccinated adults and the ability of HBsAg-RDTs to help quickly identify individuals in need of diagnosis and possible additional care at a health facility are needed.

This study has several limitations. First, the cross-sectional design limits the ability to draw causal inferences between training and other inputs and observed outcomes. Second, because survey measures relied on self-reported practices, recall-related limitations and social desirability bias may have influenced survey responses, particularly among pharmacists trained by SwipeRx and therefore more aware of their involvement in HBV testing and related care at the pharmacy. Third, the authors recognize the selection bias inherent in the category defined as “engaged pharmacies”—given that pharmacists working at these pharmacies may represent different levels of motivation or capacity relevant to HBV service provision compared to other pharmacists. Lastly, cost data related to training, coaching and other aspects of pharmacy engagement described in the manuscript were not included in the analysis and therefore, the financial scalability of the intervention was not formally evaluated.

## Conclusions

Community pharmacies in urban and rural areas represent a promising channel to increase HBV case finding through pharmacist screening with finger-prick, blood-based HBsAg-RDTs. Technology can be used to scale pharmacy professional training and post-training engagement to motivate trained pharmacists to procure and use HBsAg-RDTs to find and refer positive cases for confirmatory diagnosis and treatment. Ensuring HBsAg-RDTs pack size and contents are designed for use outside of laboratory-equipped health facilities, affordable price, and community awareness about HBV risks and benefits of community-level screening all have the potential to facilitate HBV case finding through pharmacies in Vietnam and similar country contexts. Future program and research investments should include scaling the model involving tech-enhanced pharmacies and using rigorous study designs to assess impact and cost-effectiveness.
